# High‐throughput sequencing revealed a novel SETX mutation in a Hungarian patient with amyotrophic lateral sclerosis

**DOI:** 10.1002/brb3.669

**Published:** 2017-03-15

**Authors:** Kornélia Tripolszki, Dóra Török, David Goudenège, Katalin Farkas, Adrienn Sulák, Nóra Török, József I. Engelhardt, Péter Klivényi, Vincent Procaccio, Nikoletta Nagy, Márta Széll

**Affiliations:** ^1^ Department of Medical GeneticsUniversity of SzegedSzegedHungary; ^2^ Département de Biochimie et GénétiqueIBS‐CHU AngersAngers Cedex 9France; ^3^ MTA SZTE Dermatological Research GroupUniversity of SzegedSzegedHungary; ^4^ Department of NeurologyUniversity of SzegedSzegedHungary

**Keywords:** ALS4, motor neuron disease, p.N264S missense mutation, *SETX* gene, targeted sequencing

## Abstract

**Background:**

Amyotrophic lateral sclerosis (ALS) is a neurodegenerative disease characterized by the degeneration of the motor neurons. To date, 126 genes have been implicated in ALS. Therefore, the heterogenous genetic background of ALS requires comprehensive genetic investigative approaches.

**Methods:**

In this study, DNA from 28 Hungarian ALS patients was subjected to targeted high‐throughput sequencing of the coding regions of three Mendelian ALS genes: *FUS, SETX*, and *C9ORF72*.

**Results:**

A novel heterozygous missense mutation (c.791A>G, p.N264S) of the *SETX* gene was identified in a female patient presenting an atypical ALS phenotype, including adult onset and lower motor neuron impairment. No further mutations were detected in the other Mendelian ALS genes investigated.

**Conclusion:**

Our study contributes to the understanding of the genetic and phenotypic diversity of motor neuron diseases (MNDs). Our results also suggest that the elucidation of the genetic background of MNDs requires a complex approach, including the screening of both Mendelian and non‐Mendelian genes.

## 1 Introduction

Amyotrophic lateral sclerosis (ALS; ORPHA803), also known as “Lou Gehrig's disease”, is a clinically heterogeneous group of neurodegenerative disorders characterized by the death of motor neurons in the brain, brainstem, and spinal cord resulting in fatal paralysis (Morrison & Harding, 1994). Approximately, 90% of ALS cases are sporadic and the remaining 10% are familial (Valdmanis & Rouleau, 2008). The lifetime risk for developing the disease is approximately 1/400 (Johnston et al., 2006). Due to the complex genetic background of the disease, the underlying disease‐causing variant is rarely established for individual cases (Kenna et al., 2013). To date, 126 genes have been implicated in ALS (Abel, Powell, Andersen, & Al‐Chalabi, 2012).

The most common mutants identified for the Mendelian forms are located in the *superoxide dismutase 1 (SOD1)* gene, which accounts for approximately 5% of the ALS forms (Andersen et al., 2007). Mutations in the *senataxin (SETX)* gene have been identified at a lower frequency than in the *SOD1* gene in ALS*. SETX* encodes a helicase protein involved in DNA repair and RNA production. Homozygous or compound heterozygous *SETX* mutations are associated with the development of autosomal recessive ataxia with oculomotor apraxia type 2 (AOA2; Anheim et al., 2009). In addition, heterozygous *SETX* mutations have been associated with the autosomal dominant form of juvenile‐onset ALS (ALS4; Chen et al., 2004). The *fused in sarcoma (FUS)* gene is also associated with the Mendelian forms of ALS. *FUS* encodes a nucleoprotein that functions in DNA and RNA metabolism. The *chromosome 9 open reading frame 72 (C9ORF72)* has also been identified as a disease‐causing gene of Mendelian ALS forms (Pearson et al., 2011). A heterozygous hexanucleotide (GGGGCC) repeat expansion located between the noncoding exons 1a and 1b of *C9ORF72* has been recently identified in patients with FTD and/or ALS (DeJesus‐Hernandez et al., 2011; Renton et al., 2011).

Considering the onset, the symptoms, and the course of the disease, our aim was to establish the frequencies of mutations in the coding regions of the *SETX*,* FUS*, and *C9ORF72* genes in Hungarian patients (*n* = 28) that did not carry mutations in the *SOD1* gene. The investigated patients were also negative for the *C9ORF72* hexanucleotide repeat expansion.

## 2 Patients and Methods

### 2.1 Investigated individuals

The investigated patients (*n* = 28) participated in this study were recruited from the Department of Neurology, University of Szeged, Szeged, Hungary. All patients fulfilled the revised El Escorial and the Awaji‐shima criteria for ALS (de Carvalho & Swash, 2009; Ludolph et al., 2015). According to the revised El Escorial criteria, the lower motor neuron disease (MND; progressive muscular atrophy) is determined as one of the “restricted phenotypes” of ALS; therefore, one patient with only lower motor neuron involvement was also diagnosed as ALS. All patients and age‐ and sex‐matched healthy controls (*n* = 50) were of Hungarian ancestry. The investigation was approved by the Internal Ethical Review Board of the University of Szeged. Written informed consent was obtained from patients and healthy controls, and the study was conducted according to the Principles of the Declaration of Helsinki.

### 2.2 Next‐generation sequencing

Genomic DNA was isolated from blood using the DNeasy Blood and Tissue kit (QIAGEN, Godollo, Hungary). Amplicons (*n* = 56) were designed (range 519–704 bp; mean: 612 bp) to cover the coding regions and the flanking introns of the investigated *FUS*,* SETX*, and *C9ORF72* genes, and an amplicon library was prepared according to the *Amplicon Library Preparation Manual* for the Roche Junior 454 next‐generation sequencing system (Roche, Budaörs, Hungary). Amplicons were purified with the Agencourt AMPure XP kit (Beckman Coulter, Budapest, Hungary), quantified with the Quant‐iT PicoGreen Assay (Life Technologies, Budapest, Hungary), diluted separately to 1 × 10^7^ molecules/μl and pooled. Emulsion PCR and next‐generation sequencing were performed according to the manufacturers’ protocols (Roche).

### 2.3 Bioinformatic analysis

To improve the efficiency of the Roche pipeline, composed of the Roche 454 GS Reference Mapper for mapping (on UCSC human reference genome hg19) and Amplicon Variant Analyzer (v2.5p1) for variant calling, an additional in‐house pipeline was used. Sequencing reads were aligned to the reference genome (UCSC hg19) using Roche 454 GS Reference Mapper and Amplicon Variant Analyzer (version 2.5p1). BAM files were converted to FASTQ and realigned to hg19 using Bowtie2 V.2.2.6 (Langmead & Salzberg, 2012). Samtools V.0.1.19 and Python V.2.7.11 were used for additional file handling. Variant calling was performed combining GATK Unified Genotyper (Genome Analysis Tool Kit v.3.4; McKenna et al., 2010) and Platypus (v.0.8.1; Rimmer et al., 2014). The annotation and the prioritization steps were executed with ANNOVAR (15.06.07; Wang, Li, & Hakonarson, 2010). Finally, the target region reads depth was controlled using BEDTools (v.2.25.0; Quinlan & Hall, 2010) and the alternative allele frequency was computed using pysamstats (v.0.24.2; https://github.com). Candidate variants were filtered according to read depth, allele frequency, and prevalence in genomic variant databases such as ExAc (v.0.3), ClinVar, Kaviar (including 1,000 g‐phase3, dbSNP146; Glusman, Caballero, Mauldin, Hood, & Roach, 2011; Landrum et al., 2016). Variant prioritization tools (PolyPhen2, SIFT, LRT, MutationTaster, Mutation Assessor) were used to predict the functional impact and then focused on variants with predicted deleterious consequences (nonsense SNVs, frameshift indels, essential splice variants, and complex indels). The putative effect on splicing efficiency was predicted using the Human Splicing Finder (Desmet et al., 2009). The alignments were visualized with IGV V.2.0.34. (Robinson et al., 2011). All the identified disease‐causing candidate variants were confirmed by direct sequencing.

## 3 Results

Our next‐generation sequencing approach did not detect mutations in the investigated coding regions of the *FUS* and *C9ORF72* genes.

**Figure 1:**
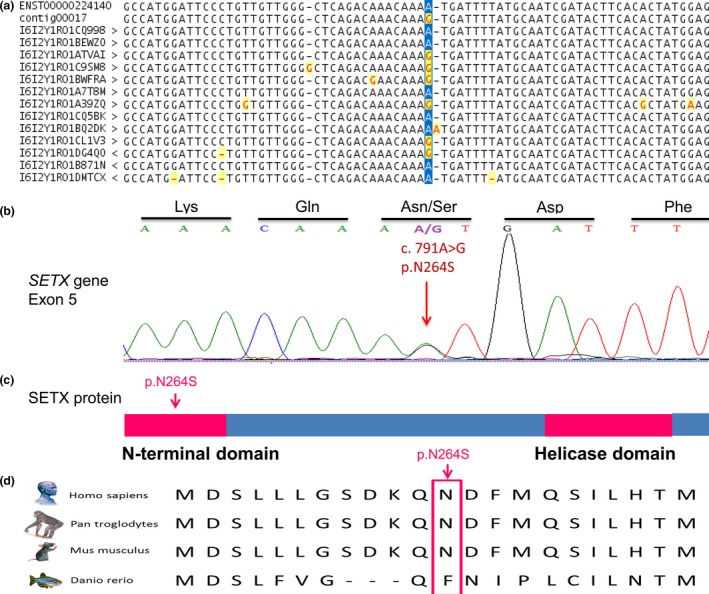
A novel mutation in the *SETX* gene. (a) Next‐generation sequencing identified the novel heterozygous missense *SETX* mutation. (b) The presence of the mutation was confirmed using direct sequencing. (c) The mutation is located within the N‐terminal domain of the encoded protein. (d) The region of the mutation is highly conserved in mammals

We identified a novel heterozygous missense mutation (c.791A>G, p.N264S) of the *SETX* gene (Figure 1a) in a Hungarian female patient. This patient presented with a lower MND phenotype, which started with an unsteady gait at the age of 65. Electroneurography revealed motor axonal loss in the right tibial and peroneal nerves with spared sensory innervation. The electromyography indicated subacute and chronic signs of denervation and reinnervation. One year later, follow up examinations indicated that denervation had spread to several muscles of both lower extremities, and the clinical and electrophysiological signs of denervation appeared in the muscles of the upper extremities. At the time blood was obtained, the interosseous muscles were wasted, dysarthria and dysphagia had developed with diminished gag reflex and lack of deep tendon reflexes were observed all over the body. Muscle strength was generally 3/5 in the upper extremities and 2/5 in the lower extremities. Sensory deficit was not observed and the extraocular muscles were not involved in the disease process. The external sphincter muscles were also spared. During the 3.5 year‐course of the disease, corticospinal or corticobulbar signs were not detected with physical examination and with magnetic resonance images of the central nervous system. Progressive weakness was observed. Other diseases causing similar symptoms were all excluded (thyroid and parathyroid diseases, paraneoplastic syndromes, gammopathies, multifocal motor neuropathy with ganglioside antibodies). Ataxia and oculomotor apraxia were not noted in the follow up examinations. The cerebrospinal fluid proved to be normal. The patient passed away at the age of 69 and the cause of death was determined to be respiratory failure due to weakness of the respiratory muscles. No other members of the family were affected. The autopsy revealed a decreased number of motor neurons in the spinal cord and diminished number of axons in the ventral roots. Hyaline inclusions were noted in the remaining spinal motor neurons. Histological examination of the striated muscles (proximal and distal in the extremities, in the diaphragm and in the tongue) revealed signs of neurogenic atrophy. There were no signs of degeneration in the corticospinal tracts. The motor cortex remained intact, and other parts of the brain (temporal and parietal lobes, hippocampus, cerebellum, thalamus, pons, and the medulla) seemed to be normal except some cell loss in the nuclei of the hypoglossal and facial nerve. Together with the clinical symptoms, the disease course and the autopsy findings confirmed the diagnosis of an atypical ALS form with lower motor neuron involvement.

The novel c.791A>G, p.N264S *SETX* mutation identified in this study was confirmed with direct sequencing (Figure 1b). SIFT, Polyphen, and Mutation Taster analyses determined this variant as a damaging, disease‐causing mutation. The mutation was absent in the patient's clinically healthy 45‐year‐old son and in the age‐ and gender‐matched healthy control individuals. The heterozygous p.N264S mutation affects the N‐terminal region of the SETX protein (Figure 1c) and is located in a region which is conserved among mammals (Figure 1d). According to the results of the Human Splicing Finder online prediction tool, the c.791A>G mutation could result in activation of an exonic cryptic donor site, creation of an exonic silencer site or alteration of an exonic enhancer site.

## 4 Discussion

**Figure 2:**
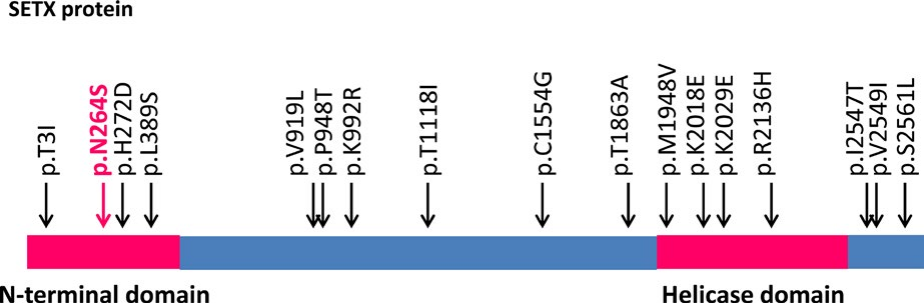
*SETX* mutations associated with ALS4. All identified disease‐causing *SETX* mutations implicated in ALS4 are heterozygous missense. Other types of mutations have not been detected in ALS4. The identified mutations are located in coding regions and distributed nearly evenly on the encoded protein. Mutation hot spots have not been detected. Mutations are located both within the N‐terminal and helicase domains and located outside these regions, suggesting that these regions have as yet unidentified pivotal biological functions

In this study, we investigated three Mendelian disease genes (*SETX, FUS*, and *C9ORF72*) in 28 Hungarian sporadic ALS patients. We identified a novel, disease‐causing heterozygous missense mutation (p.N264S) in the *SETX* gene. To date, only 17 pathogenic *SETX* mutations (Figure 2) have been implicated in the development of the autosomal dominant juvenile form, ALS4. The typical phenotype, present in the majority of the ALS4 patients, presents as a combination of lower and upper motor neuron impairments (Avemaria et al., 2011; Hirano et al., 2011; Saracchi et al., 2014).

Originally, the spectrum of adult MNDs included ALS with the combined degeneration of upper (cortical) and lower (pontobulbar and spinal) motor neurons, primary lateral sclerosis (PLS) with only upper motor neuron lesion and lower MND with the damage of pontobulbar and/or spinal motor neurons (Victor & Ropper, 2000). In a recent work, Finsterer and Burgunder (2014) suggested that ALS can manifest clinically as a continuum ranging from exclusive impairment of the upper motor neurons to exclusive impairment of the lower motor neurons. Exclusive impairment of the upper motor neurons can manifest in PLS characterized by spastic paresis of the striated muscles without atrophy and denervation, increased deep tendon reflexes, pathological reflexes designating upper motor neuron lesion (Finsterer & Burgunder, 2014). Exclusive impairment of the lower motor neurons can contribute to the development of lower MNDs observed as atrophy of the striated muscles of the body due to degeneration of lower motor neurons with increasing weakness, decreased muscle tone, electrophysiological signs of denervation (fasciculations, fibrillations), lack of the deep tendon reflexes and, finally, inevitable death from respiratory failure (Finsterer & Burgunder, 2014; Victor & Ropper, 2000).

The identified novel p.N264S SETX mutation is associated with an unusual ALS phenotype characterized by lower motor neuron impairment only. Our results further support the accepted view that ALS and lower MNDs are not different disease entities, but that, instead, they are clinical variants of the same disease spectrum. The phenotypic variation can be explained by yet unidentified genetic modifiers, environmental and/or life style factors. The unusual phenotype observed in the reported Hungarian patient is not unique in the literature of pathogenic heterozygous *SETX* mutations. A similar atypical phenotype has been described in a Chinese patient carrying the p.T1118I heterozygous missense mutation of the *SETX* gene (Zhao et al., 2009). Although the p.N264S mutation is located within the N‐terminal region of the protein and the p.T1118I mutation is located in a region of yet unknown function, both of these mutations result in the development of the same unusual ALS4 phenotype. Further studies are needed to elucidate the underlying mechanism responsible for the observed unusual phenotypes associated with the heterozygous p.N264S and p.T1118I missense mutations of the *SETX* gene (Table 1).

**Table 1:** Clinical features of the atypical ALS4 phenotype

SETX mutation	Amino acid substitution	Ethnic origin	Age of onset	Lower motor neuron sign	Upper motor neuron sign	Bulbar sign	Sensory impairment	Reference
c.791A>G	p.N264S	Hungarian	65	Present	Absent	Present	Absent	This study
c.3353C>T	p.T1118I	Chinese (Han)	42	Present	Absent	Present	Absent	Zhao et al. (2009)

The 17 known heterozygous missense mutations of the *SETX* gene are located in exons 1, 5, 8, 11, 12, 13, 17, and 24 (Table 2). Four of the 17 mutations are located within the helicase domain of the SETX protein (Chen et al., 2004; Hirano et al., 2011; Kenna et al., 2013; Saracchi et al., 2014). In AOA2, homozygous or compound heterozygous missense mutations located within the helicase domain are generally associated with less severe phenotypes than the mutations affecting other regions of the protein (Anheim et al., 2009; Moreira et al., 2004). A similar genotype–phenotype association has not been observed for the heterozygous missense mutations associated with ALS4. However, in ALS4, mutations impairing the region of the helicase domain are associated with cortical and spinal motor neuron impairment, whereas others located outside of this region can lead to the development of either spinal or bulbar motor neuron involvement (Table 2).

**Table 2:** Clinical manifestations of *SETX* mutations in ALS4

SETX mutation	Amino acid substitution	Affected exon	Impaired region of the protein	Age of onset	Affected sites	Reference
c.8C>T	p.T3I	1	N‐terminal domain	8	Spinal	Chen et al. (2004)
c.791A>G	p.N264S	5	N‐terminal domain	65	Both	This study
c.814C>G	p.H272D	5	N‐terminal domain	66	Bulbar	Kenna et al. (2013)
c.1166T>C	p.L389S	8	N‐terminal domain	17	Spinal	Chen et al. (2004)
				24	Spinal	Avemaria et al. (2011)
c.2755G>C	p.V919L	8	Unknown function	68	Both	Kenna et al. (2013)
rc.2842C>A	p.P948T	8	Unknown function	58	Bulbar	Kenna et al. (2013)
c.2975A>G	p.K992R	8	Unknown function	78	Bulbar	Kenna et al. (2013)
c.3353C>T	p.T1118I	8	Unknown function	42	Both	Zhao et al. (2009)
c.4660T>G	p.C1554G	8	Unknown function	6	Spinal	Hirano et al. (2011)
c.5587A>G	p.T1863A	11	Unknown function	61	Other	Kenna et al. (2013)
c.5842A>G	p.M1948V	12	Helicase domain	47	Spinal	Kenna et al. (2013)
c.6052A>G	p.K2018E	13	Helicase domain	54	Spinal	Saracchi et al. (2014)
c.6085C>G	p.K2029E	13	Helicase domain	35	Spinal	Hirano et al. (2011)
c.6407G>A	p.R2136H	17	Helicase Domain	6	Spinal	Chen et al. (2004)
c.7640T>C	p.I2547T	24	Unknown function	30	Spinal	Hirano et al. (2011)
c.7645G>A	p.V2549I	24	Unknown function	64	Spinal	Kenna et al. (2013)
c.7682C>T	p.S2561L	24	Unknown function	84	Spinal	Kenna et al. (2013)

It has also been demonstrated that, in AOA2, missense mutations affecting the N‐terminal or the helicase domain of SETX cause phenotypes similar to those caused by deletions, nonsense, or frameshift mutations (Chen et al., 2006). However, only missense *SETX* mutations have been implicated in the development of ALS4, to date (Avemaria et al., 2011; Hirano et al., 2011; Kenna et al., 2013; Saracchi et al., 2014); deletions, nonsense, frameshift, or other types of mutations have not yet been detected for this disease (Table 2).

Our study further widens the geographic range for the origin of disease‐causing heterozygous missense mutations of the *SETX* gene, which have already been implicated in ALS in patients from different countries (Avemaria et al., 2011; Hirano et al., 2011; Kenna et al., 2013; Saracchi et al., 2014). To the best of our knowledge, our study is the first demonstrating a novel *SETX* mutation in the Hungarian ALS population (Table 2).

In this study, we did not identify any mutations in the coding regions of the *C9ORF72* gene. These results correlate well with the data reported in the literature; there are currently no reports of disease‐causing mutation in the coding regions of the *C9ORF72* gene (Kenna et al., 2013; Koppers et al., 2013).

No mutations in *FUS*, another Mendelian gene associated with ALS, were detected in the Hungarian patients. *FUS* mutations have been detected in two regions of the gene that consequently affect the encoded protein: approximately one‐third are located within exon 3 and 6 and impair the glutamine‐glycine‐serine‐tyrosine‐rich and the arginine‐glycine‐glycine‐rich domains of the protein. Two‐thirds of the mutations are located within the region including exons 12–15, which encodes a zinc finger and arginine‐glycine‐glycine‐rich domains (Shang & Huang, 2016). On the basis of our results, we hypothesize that *FUS* mutations in the Hungarian ALS population might be very rare. This hypothesis is supported by the literature, as *FUS* mutations have been reported to contribute to the development of the disease in approximately 0.5% of the patients (Kenna et al., 2013).

On the basis of our results and the results of previous studies, we also emphasize that the genetic screening of the Mendelian ALS‐associated genes might not elucidate the causative genetic variant in the majority of ALS cases (Cirulli et al., 2015; Kenna et al., 2013). The genetic heterogeneity of ALS is extremely complex: rare mutations of Mendelian genes and common variants of non‐Mendelian genes can also contribute to the development of the disease (Abel et al., 2012). Our comprehensive study adds novel data to the genetic and phenotypic diversity of ALS and indicates that complex approaches, high‐throughput methods, and large‐scale studies are needed to understand of the genetic heterogeneity of this disease.

## Conflict of Interest

No potential conflict of interest was reported by the authors.
